# Neuroimaging and Neuropsychological Outcomes Following Clinician-Delivered Cognitive Training for Six Patients With Mild Brain Injury: A Multiple Case Study

**DOI:** 10.3389/fnhum.2020.00229

**Published:** 2020-06-24

**Authors:** Amy Lawson Moore, Dick M. Carpenter, Randolph L. James, Terissa Michele Miller, Jeffrey J. Moore, Elizabeth A. Disbrow, Christina R. Ledbetter

**Affiliations:** ^1^Department of Psychology, Gibson Institute of Cognitive Research, Colorado Springs, CO, United States; ^2^College of Education, University of Colorado Colorado Springs, Colorado Springs, CO, United States; ^3^True Life Medicine, Woodland Park, CO, United States; ^4^School of Nursing, Colorado State University-Pueblo, Pueblo, CO, United States; ^5^Department of Neurology, Louisiana State University Health Sciences Center, Shreveport, LA, United States; ^6^Louisiana State University Health Sciences Center, Center for Brain Health, Shreveport, LA, United States; ^7^Department of Neurosurgery, Louisiana State University Health Sciences Center, Shreveport, LA, United States

**Keywords:** cognitive training, cognitive rehabilitation, MRI, LearningRx, brain training, mild TBI, concussion, brain injury

## Abstract

Nearly half of all mild brain injury sufferers experience long-term cognitive impairment, so an important goal in rehabilitation is to address their multiple cognitive deficits to help them return to prior levels of functioning. Cognitive training, or the use of repeated mental exercises to enhance cognition, is one remediation method for brain injury. The primary purpose of this hypothesis-generating pilot study was to explore the statistical and clinical significance of cognitive changes and transfer of training to real-life functioning following 60 h of Brain Booster, a clinician-delivered cognitive training program, for six patients with mild traumatic brain injury (TBI) or non-traumatic acquired brain injury (ABI). The secondary purpose was to explore changes in functional connectivity and neural correlates of cognitive test gains following the training. We used a multiple case study design to document significant changes in cognitive test scores, overall IQ score, and symptom ratings; and we used magnetic resonance imaging (MRI) to explore trends in functional network connectivity and neural correlates of cognitive change. All cognitive test scores showed improvement with statistically significant changes on five of the seven measures (long-term memory, processing speed, reasoning, auditory processing, and overall IQ score). The mean change in IQ score was 20 points, from a mean of 108 to a mean of 128. Five themes emerged from the qualitative data analysis including improvements in cognition, mood, social identity, performance, and Instrumental Activities of Daily Living (IADLs). With MRI, we documented significant region-to-region changes in connectivity following cognitive training including those involving the cerebellum and cerebellar networks. We also found significant correlations between changes in IQ score and change in white matter integrity of bilateral corticospinal tracts (CST) and the left uncinate fasciculus. This study adds to the growing body of literature examining the effects of cognitive training for mild TBI and ABI, and to the collection of research on the benefits of cognitive training in general.

**Clinical Trial Registration:** www.ClinicalTrials.gov, identifier NCT02918994.

## Introduction

Cognitive deficits are common following brain injury, especially in working memory, attention, and processing speed – skills that are critical for information processing, decision making, and learning. With rehabilitation, many patients suffering from brain injury experience improvement in cognitive functions as well as quality of life struggles (Tsaousides and Gordon, 2009; Iaccarino et al., 2015). However, nearly half of all mild brain injury sufferers experience long-term cognitive impairment (McInnes et al., 2017). Therefore, an important goal in rehabilitation is to address the multiple cognitive deficits seen in sufferers of mild brain injury to help them return to prior levels of functioning.

### Cognitive Training for TBI

Cognitive training is the use of repeated mental exercises to enhance cognition. The literature is replete with studies examining the efficacy of several cognitive training methods across diagnostic categories, particularly for moderate to severe traumatic brain injury (TBI) (Ragnarsson et al., 1999; Hallock et al., 2016; Cicerone et al., 2019). For example, Phillips et al. (2016) examined computerized memory training for children with moderate to severe TBI, finding greater scores in reading achievement and significant gains in working memory but no significant changes in attention. Sharma et al. (2017) examined the feasibility of a 12 weeks, self-administered digital cognitive training program for patients with moderate to severe TBI, reporting on no efficacy results but finding a 30% attrition rate and only a 42.6% completion rate. Another study with 17 pediatric TBI participants who completed the Canadian *Ready! Set? Let’s Train!* (RST) program revealed improvements in working memory, inhibition, and cognitive flexibility (Seguin et al., 2018). Ledbetter et al. (2017) reported clinically significant change across cognitive skills following 90 h of clinician-delivered cognitive training to soldiers with moderate to severe TBI. However, there is a notable gap in the research regarding cognitive training interventions for the more subtly nuanced deficits inherent in mild TBI and mild non-traumatic ABI. This study seeks to help fill that gap.

### Magnetic Resonance Imaging (MRI) of Cognitive Training for Brain Injury

Pathological changes in neural structure and network function following TBI is well established by MRI in research (Sharp et al., 2011; Croall et al., 2014; Sharp et al., 2014; Han et al., 2016). There is also robust history of utilizing resting state functional MRI (fMRI) to explore intervention-related changes in functional connectivity, including changes following the use of cognitive behavioral therapy (Chattopadhyay et al., 2017), electroconvulsive therapy (Leaver et al., 2016), golf training (Bezzola et al., 2012), music training (Imfeld et al., 2009), cognitive training (Deng et al., 2019), and multiple educational interventions (Barquero et al., 2014). Researchers have prolifically published activation studies of brain networks during cognitive training tasks (Floyer-Lea and Matthews, 2005; Klingberg, 2010; Jolles et al., 2011; Finc et al., 2019), as well as studies examining gray matter change following cognitive training (Takeuchi et al., 2013; Catharine et al., 2019). Assessment of functional connectivity in moderately to severely brain injured patients before and after cognitive training has also been well-documented. For example, Han et al. (2017) noted connectivity changes as indicators of brain plasticity resulting from cognitive training and in a follow up study (Han et al., 2018b) utilized resting state fMRI to examine the impact of strategy-specific cognitive training on neural networks. They noted reduction in depressive symptoms that significantly correlated with reduced connectivity in the right VLPFC, right APFC, right DPFC1, and right DPFC2 of 57 adults an average of 9 years post-brain injury. Han et al. (2020) also published conclusive findings of modular network reorganization in TBI patients who underwent cognitive training.

The association between cognitive performance and white matter integrity has been well-documented for more than a decade in mild brain injury (Niogi et al., 2008; Hurtubise et al., 2020), and the influence of cognitive training on white matter changes correlating with cognition improvement has been documented in rats with mild TBI (Braeckman et al., 2019). However, there is a paucity of research using MRI functional and structural connectivity measures to explore changes following clinician-delivered cognitive training in young adults with mild brain injury. Given this gap in the literature, the purpose of the current study was first to explore the statistical and clinical significance of cognitive changes and transfer of training to real-life functioning following 60 h of clinician-delivered cognitive training for six patients with mild brain injury. Then, we wanted to explore if neuroimaging could provide insights into the mechanism of change from this method of cognitive training including changes in functional connectivity and neural correlates of cognitive test gains.

## Materials and Methods

### Participants

The sample for the current study includes six patients recovering from mild brain injury with diverse etiology. There were four males and two females, five Caucasian and one Asian-American, ranging in age from 15 to 35 (mean = 22.7). Patients were recruited through physicians and mental health providers in southern Colorado. Inclusion criteria were consistent with *The Mild Traumatic Brain Injury Committee of the Head Injury Interdisciplinary Special Interest Group of the* American Congress of Rehabilitation Medicine (1993) including loss of consciousness less than 30 min, post-traumatic amnesia less than 24 h, and altered mental state at the time of injury or a documented history of concussion or mild non-TBI meeting the former criteria. In addition, patients must have had cognitive symptoms lasting more than 12 months post-injury to ensure patients were beyond the spontaneous recovery period. All patients in the sample provided informed consent at the onset of the study.

### Study Design

The current study used a case study design across multiple cases. The multiple case study method (Yin, 2014) enables in-depth evaluation of an approach to care and, therefore, has a long history of use in medical and psychological research. The design enabled us to collect preliminary evidence from a variety of sources and to examine similarities and differences in the effects of the intervention on a diverse set of cases (Mills et al., 2010) to support a larger controlled study. The study was approved by the Institutional Review Board (IRB) at Gibson Institute of Cognitive Research under Approval Number 10122016, which certified the study met the criteria for Subpart A Basic HHS Policy of Protection of Humans Research Subjects of the Code of Federal Regulations, Title 45, Part 46.

### Intervention

**FIGURE 1:**
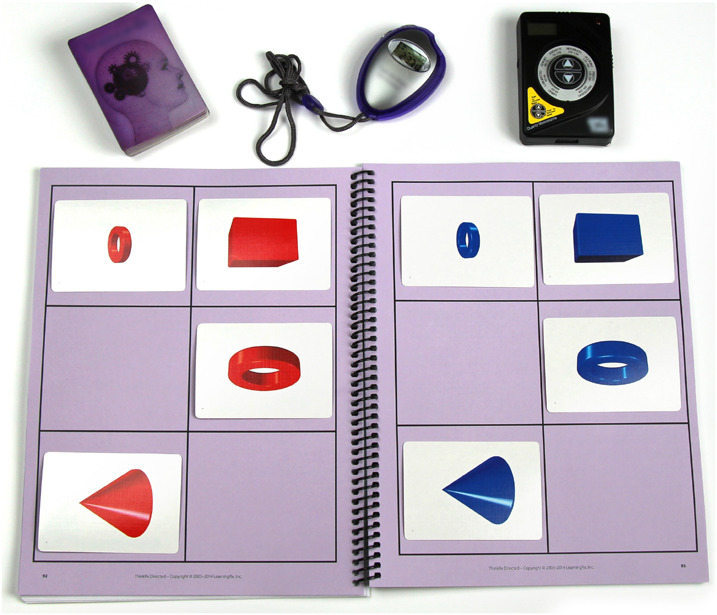
Example of a training task.

The cognitive training methodology used in the current study is called *Brain Booster* (Gibson et al., 2013) by LearningRx, a global network of cognitive training centers. The 132-page curriculum consists of 16 basic mental training exercises with 520 variations sequenced in order of difficulty and complexity. Each participant attended three 90 min training sessions each week for about 14 weeks. Certified cognitive trainers delivered the program, monitored by a doctoral level psychologist to ensure treatment fidelity for the study. Trainers tracked the progress of each participant using a web-based task flow program called the Training Management System (TMS). Consistent with the LearningRx training model (see Carpenter et al., 2016), *Brain Booster* is delivered one-on-one, face-to-face using a variety of hands-on manipulatives including shape and number cards, tangrams, a metronome, and a timer. The training exercises target the development and remediation of multiple cognitive constructs including working memory, long-term memory, processing speed, fluid reasoning, visual processing, auditory processing, and attention. No training task is designed to target one skill in isolation, however. Each task simultaneously addresses multiple cognitive skills. For example, the Memory Match (Figure 1) training task targets the primary skill of visual working memory, but also targets processing speed and sustained attention along with visual discrimination and visual span.

The human-delivery format is a unique attribute of the methodology and departs from the abundance of digital training products described in the extant research. The trainer facilitates an intense training session by using rapid pacing for the entire 90 min, transitioning from one task to the next without breaks. Nearly all training procedures are paced using a metronome to increase the intensity of tasks. The participant must respond on each beat or on every other beat to the metronome set at 85–160 beats per minute, which eliminates wait time and forces rapid responses to the stimuli presented. Many of the tasks also have variations that use a timer and require participants to complete a certain number of items within a short time limit. Deliberate distractions such as talking, singing, stating incorrect responses on the tasks, or telling a joke are added by the trainer to mimic stimulus-rich environments in which we live and work and that force patients to attend to only the relevant stimuli.

The dynamic feedback provided by human trainers is another unique aspect of this training method. Trainers coach the participants through each task, adding complexity to challenge them, reducing intensity when appropriate, and encouraging them as they master training procedures. This model of interaction is supported by Bandura’s (1994) theory of self-efficacy – the belief in one’s ability – by providing all four sources of self-efficacy development: modeling, mastery experiences, verbal persuasion, and guided response to stress.

### Outcome Measures

#### Woodcock Johnson IV Tests of Cognitive Abilities (WJ IV)

The WJ IV Tests of Cognitive Abilities (Schrank et al., 2014) measures individual cognitive skills and composite general intellectual ability (GIA) in comparison to a normative group. We selected six primary cognitive skill subtests and reported the composite GIA score generated by Tests 1–7 in the core battery. A description of the cognitive outcome measures follows.

*Number Series* is a test of fluid reasoning – specifically, quantitative, deductive, and inductive reasoning. This test requires the examinee to supply the missing number from a sequence of numbers following a mathematical pattern. This test has a median reliability of 0.91 and a mean reliability of 0.90 for the ages of the patients in the study.

*Verbal Attention* is a test of short-term working memory and sustained attention that requires the examinee to listen to lists of animals and numbers and then answer a question based on each sequence of information. This test has a median reliability of 0.86 and a mean reliability of 0.87 for the ages of the patients in the study.

*Letter-Pattern Matching* measures the speed at which the examinee can make visual symbol discriminations among a series of letter patterns. This test evaluates processing speed and broad attention skills. This test has a median reliability of 0.88 and a mean reliability of 0.90 for the ages of the patients in the study.

*Phonological Processing* assesses the examinee’s word retrieval abilities using phonological cues. This subtest assesses auditory processing and automatic retrieval skills. This test has a median reliability of 0.84 and a mean reliability of 0.85 for the ages of the patients in the study.

*Story Recall* measures listening ability and reconstructive long-term memory. The task requires the examinee to recall details of increasingly complex stories. This test has a median reliability of 0.93 and a mean reliability of 0.92 for the ages of the patients in the study.

*Visualization* measures two aspects of visual-spatial processing involving visual feature detection and mental rotation of objects. One component requires the examinee to identify two or three pieces that form a completed target shape. The other part requires the examinee to identify rotated block configurations that correspond to a target configuration. This test has a median reliability of 0.85 and a mean reliability of 0.84 for the ages of the patients in the study.

*General Intellectual Ability (GIA)*, or IQ score, represents a measure overall intelligence. It is a weighted composite of the prior six subtests in addition to an Oral Vocabulary test. This cluster has a median reliability of 0.97 and a mean reliability of 0.96 for the ages of the patients in the study.

#### TBI Patient Competency Rating Scale (PCRS)

The Patient Competency Rating Scale (PCRS) was given to each patient and a family member. It evaluates perceived function in three areas: activities of daily living (ADLs), interpersonal and emotional function, and cognitive abilities. It is a 30-item 5-point Likert survey with a reliability of 0.97 for patients and 0.92 for relatives (Kolakowsky-Hayner, 2010). Questions pertaining to ADLs ask about problems with dressing, personal hygiene, preparing meals, doing laundry, and managing finances, for example. Items pertaining to interpersonal and emotional functioning ask about things like handling arguments, controlling crying, showing affection, participating in group activities, recognizing when you have upset someone, controlling laughter, or feeling depressed. Items pertaining to cognition ask about things like scheduling activities, understanding instructions, remembering appointments, driving a car, concentrating on work activities, and remembering names.

#### Magnetic Resonance Imaging

Pre- and post-intervention Magnetic Resonance Imaging (MRI) was used to examine changes in resting state connectivity and correlation of changes in white matter tracts to changes in overall IQ score. Imaging was performed using a single 3 Tesla Siemens Skyra (Erlangen, Germany) MRI system. At each exam we acquired a high-resolution T1-weighted anatomical scan, a resting state T2^∗^-weighted BOLD (blood oxygen level dependent) scan, and a diffusion weighted scan. To acquire high-resolution T1-weighted anatomical images, we used a magnetization prepared rapid gradient echo (MP-RAGE) pulse sequence with these parameters: TE = 2.57 ms, TR = 1900 ms, 192 slices, FOV = 230 mm × 230 mm, matrix size = 512 × 512, and slice thickness = 1 mm. We acquired the T2^∗^ blood oxygenation level-dependent (BOLD) weighted functional images with a single-shot gradient echo planar imaging (EPI) sequence with these parameters: TE = 30 ms, TR = 3000 ms, slices = 36, FOV = 230 mm x 230 mm, matrix size = 128 × 128, slice thickness = 4 mm, number of acquisitions = 240, acquisition time = 12 min. PACE, an online prospective motion correction acquisition algorithm, was used for functional imaging and these motion corrected images for used in all analyses. Functional imaging was performed during the resting state with patients instructed to relax, clear their minds, and keep their eyes open. The diffusion weighted scan was acquired with a single-shot multi-focused EPI pulse sequence with these parameters: TE = 95 ms, TR = 4300 ms, *b*-value = 1000 s/mm^2^, 30 directions, slices = 27, FOV = 230 mm × 230 mm, matric size = 128 × 128, slice thickness = 4 mm, non-diffusion-weighted images = 12, acquisition time = 8 min.

#### Qualitative Interviews

Patients were interviewed three times: during intake, midway through the intervention period, and at the end of the intervention. Interviews were semi-structured with open-ended questions designed to obtain information about patient symptoms related to cognition and daily living. At intake, the research team asked, “What symptoms related to ____ are you experiencing?” including thinking and cognition, physical functioning, emotions and feelings, activities in daily life. The same questions were posed at mid and post-intervention along with the same topics inserted into the question, “What specific changes have you seen in _______ since training began?” The interviews began with casual and open-ended conversation designed to elicit a discussion about each patient’s functioning and experiences related to their brain injuries, their overall functioning before, during, and after the intervention, and their intervention experience. The interview questions were crafted specifically to ensure we obtained comments related to all four areas of functioning. We intentionally chose the term “changes” to prevent leading questions. Thus, the language was neutral, focusing on a discussion of any changes noticed by patients and families, which invited them to report either improvements or declines in functioning.

### Data Analyses

#### Individual Cognitive Assessment Data Analysis

The Woodcock Johnson IV test data for each patient were analyzed for clinically significant change and the Reliable Change Index (RCI). We used a two-step procedure to calculate the clinical significance of the test gains for the individual patients. First, we used the Jacobson-Truax Method (Jacobson and Truax, 1991) to algebraically determine the cut point of the healthy population for each construct, or the value above which a score should fall in the distribution of scores in a healthy population. The cut point for each construct is calculated with the following formula:

(M×dSD)n+(M×nSD)d/(SD+nSD)d

Where

M_d_ = study sample mean

SD_d_ = study sample standard deviation

M_n_ = Woodcock Johnson age-matched standardization sample mean

SD_n_ is the Woodcock Johnson age-matched standardization sample standard deviation.

In the second step, we determined if the magnitude of the change was statistically reliable using a RCI for each patient. The RCI indicates the change beyond what might be expected by chance due to testing instrument variability. Using the standard error of the difference in a classical measurement theory RCI formula, changes that exceed 1.96 times the standard error are not likely to occur more than 5% of the time. The formula for calculating the RCI is as follows:

X-2X/1Sdiff

where

X_1_ = participant’s Woodcock Johnson pretest standard W score

X_2_ = participant’s Woodcock Johnson post-test standard W score

S_diff_ = standard error of the difference score.

We did not conduct clinically significant change analyses on the PCRS scores for each patient because there are no published age-based norms, which are required for the calculations.

#### Group Psychometric Testing and Functional Ratings Data Analysis

In addition to analyzing clinically significant change for the individual patients, we also analyzed pre-post-differences in the Woodcock Johnson IV and PCRS test metrics across all six participants as a group. Given the small sample size, we used non-parametric tests, specifically a Wilcoxon Rank test, the non-parametric equivalent to a paired samples *t*-test. Analyses were conducted using IBM SPSS Version 24.

#### Neuroimaging Data Analysis

We performed anatomical and resting-state image pre-processing and resting-state connectivity data analysis using the CONN toolbox (RRID:SCR_009550^1^; Nieto-Castanon, 2020), a software package with tools that minimize motion artifacts and physiological noise and allows for valid interpretation of negative correlations aka anticorrelation (Whitfield-Gabrieli and Nieto-Castanon, 2012). During pre-processing, the data were realigned, slice-time corrected, normalized to MNI stereotactic space, and spatially smoothed with an 8-mm FWHM Gaussian kernel. With the CONN artifact reduction tool (ART), we first identified single functional images containing outliers in motion and global signal. Then, we defined them as nuisance parameters within first level general linear models. For ART, we used a global-signal *z*-value threshold of 5 and a subject-motion threshold of 0.9 mm. With the CONN aCompCor tool, we identified physiological and spurious sources of noise to use as nuisance parameters within first level general linear models. For aCompCor, we used each patients’ high-resolution anatomical images to segment gray matter (GM), white matter (WM) and cerebrospinal fluid (CSF), and then applied these masks to the BOLD images. The first three principle components of the BOLD time series from WM and CSF were included as nuisance parameters. Finally, we temporally band-pass filtered residual BOLD time-series (0.008 < *f* < 0.09). We generated first-level individual ROI-to-ROI correlation maps by extracting the residual BOLD time series from each ROI, and then calculating Pearson’s correlation coefficients between all ROIs (164 × 164). Correlation coefficients were transformed into Fisher’s Z scores for use in second-level group analyses. For the functional connectivity analyses, False Discovery Rate (FDR) corrections for multiple comparisons were applied using the CONN Toolbox function call *p_FDR* = *conn_fdr(p_uncorrected)* set at *p* < 0.05.

We performed anatomical and diffusion weighted image pre-processing and white matter fiber tract data analysis using longitudinal pipelines in FreeSurfer Version 6.0.0 (Reuter et al., 2012)^2^ and its TRActs Constrained by UnderLying Anatomy (TRACULA) toolbox (Yendiki et al., 2011, 2016). Freesurfer allows for the optimized analysis of longitudinal data by using anatomical images across time points to create an unbiased anatomical base template, which is then used in TRACULA along with a probabilistic atlas to automatically reconstruct a set of major white matter pathways with a point-to-point correspondence across time points. First, using the acquired anatomical image from each time point, we performed volumetric cortical reconstruction and volumetric segmentation. This processing included motion correction, deletion of non-brain tissue, automated Talairach transformation, gray and white matter segmentation, intensity normalization, automated correction of topology, surface tiling, surface inflation, and registration to a spherical atlas. Second, we utilized the resulting single time point images to create an unbiased, within-subject base template. Finally, using the base template and the diffusion data we performed reconstruction and longitudinal fractional anisotropy (FA) quantification for 17 major white matter tracts: left and right (L/R) corticospinal tracts (CST), L/R inferior longitudinal fasciculus (ILF), L/R uncinate fasciculus (UNC), L/R anterior thalamic radiation (ATR), L/R cingulum cingulate bundle (CCG), L/R cingulum angular bundle (CAB), L/R superior longitudinal fasciculus parietal bundle (SLFP), L/R superior longitudinal fasciculus temporal bundle (SLFT), and corpus callosum forceps major (FMAJ). This processing included eddy current correction, affine registration to base template, and MNI template registration. The details of these procedures and their validity are referenced on the Freesurfer website. To correct for multiple comparisons in the second-level analysis, we used the Benjamini-Hochberg procedure (Benjamini and Hochberg, 1995). We compared each individual *p*-value from the Pearson’s correlation to its Benjamini-Hochberg critical value computed using the following formula: BH = (I/m)^∗^Q where I is the correlation coefficient rank (*I* = 1–17), m is the total number of tests (*m* = 17), and Q is the false discovery rate (*Q* = 0.2). The largest *p*-value where *p* < BH is significant, and all *p*-values smaller that it are also significant.

#### Qualitative Data Analysis

Qualitative data were analyzed using an inductive approach to thematic analysis (Liu, 2016), which allows phenomenological themes to emerge and coalesce from the ground up. Qualitative comments were obtained objectively during the research process with purposeful disregard for prior interview data (Tetnowski, 2015). Researchers addressed concerns of validity and rigor by application of triangulation, objective audit, and grounded methodology (Houghton et al., 2013). Triangulation was assured through commentary collection via multiple sources (patient, patient’s relative, trainer), by various investigators (research director, trainer, test administrator), and with diverse perspectives (clinical notes from pre-intervention interviews, mid-intervention interviews and post-intervention interviews). Data were collected and recorded with impartiality and indiscriminately evaluated without bias for outcome. Data were coded at the phrase level and then themes were evaluated and clarified by two members of the research team.

## Results

### Patient Histories and Outcomes

#### Patient One

Patient One, a 35 years old female, was involved in a rear-impact motor vehicle accident (MVA), which totaled the car 2 years prior to the study. She had no loss of consciousness or visible injuries and declined transport for medical attention at the time of the accident. She was diagnosed with whiplash at an urgent care clinic the following day after presenting with neck pain and headache. She was later diagnosed with diffuse TBI with loss of consciousness less than 30 min by a neuropsychiatrist. A clinical read of the study MRI exam noted minimal bifrontal white matter hyperintensities and a cavum septum pellucidum et vergae, which may or may not been a result of trauma. In her pre-intervention intake interview, she reported visual and auditory processing deficits, memory lapses, reading struggles, and diminished conversational ability saying, “I just can’t think anymore! Can’t remember things from one minute to the next, so I lose things and forget and feel lost sometimes.” She reported lost independence, social connections, and driving ability and described seizure-like episodes triggered by visual or auditory input. Throughout the intervention period, she reported these episodes diminished until non-existent. The trainer noted her progressive ability to focus, improved memory, and mental stamina. Following 60 h of cognitive training, the patient and her spouse reported improved cognitive function, confidence, renewed social interaction, and the ability to read, drive, and cook again. She stated, “I’m less overwhelmed, not avoiding people as much; I can engage in social activities much better…read longer now than before, and I can talk on the phone now and focus on the conversation.”

#### Patient Two

Patient Two, an 18 years old female, suffered a mild TBI falling off a sled 4 years prior to the study where she suffered no loss of consciousness but was diagnosed in the emergency room with a broken wrist and concussion. She reported a subsequent inability to focus, personality changes, and social isolation. She presented with cognitive deficits, depression, and anxiety prior to the intervention. During the study period, the patient’s cognitive trainer noted improvements in attention, eye-contact, perseverance, and frustration tolerance. The patient said, “I can juggle more tasks, can actually do things on my to-do list in order of importance; less distracted by everything.” After 60 h of cognitive training, she reported improved focus and memory, and stated, “I’m more spontaneous now, more hopeful, I have friends again! Got my first job – stressful, but I can feel my working memory kicking in.” Her mother noted her improved mood and increased driving independence.

#### Patient Three

Patient Three was a 15 years old male who suffered three sports-related concussions without loss of consciousness along with dozens of sub-concussive hits to the head in sports practice and games. His mother reported a history of head-butting in early childhood and umbilical cord-related hypoxia in childbirth as well. A clinical read of the study MRI exam noted white matter hyperintensities within the frontal lobe consistent with prior head injury. He presented with cognitive deficits, impulsivity, school failure, social problems, and a previous diagnosis by a psychologist of Attention Deficit Hyperactivity Disorder (ADHD). The patient stated a desire for better school achievement and improved social identity. During the study period, the patient’s cognitive trainer noted improved concentration and memory and described the patient as “less distracted, capable of more, and works for longer periods.” The patient began setting and achieving personal goals and joined new social groups. Following 60 h of cognitive training, he reported faster schoolwork completion saying, “I can focus on stuff longer…can see the pitch earlier (in baseball) …I fight less than I used to.” The patient’s mother reported improved school performance, less arguing, and more drive. She stated, “Now he can sit for over an hour doing schoolwork; he used to get very distracted. His goals in life have changed…he’s focused on the future and not so much the moment.”

#### Patient Four

Patient four was a 19 years old male who had suffered five sports-related concussions in high school all with loss of consciousness less than 5 min. The last injury occurred 15 months prior to study enrollment. Concussions were diagnosed by a sports medicine physician and confirmed by a neurologist who added post-concussion syndrome to the diagnosis. The patient reported problems with memory, focus, visual processing, and auditory processing, as well as low motivation and school achievement, depression, and isolation. He stated, “After the 4th concussion…I have trouble like remembering things, like people’s names, remembering what I need to do, following conversation for even more than like 30 seconds.” At the mid-training interview, the patient’s parents reported he was, “more assertive cognitively in conversation, lucid and clear-minded.” Following 60 h of training, the cognitive trainer noted more confidence, perseverance, and better outlook. The patient reported improvements with memory, attention, visual and auditory processing, and processing speed, with better school performance and quality of life. He stated, “I’ve noticed I haven’t lost or misplaced things as much. I feel more confident in conversations – easier to articulate things; helped me with friends.”

#### Patient Five

Patient Five was a 22 years old male with an acquired brain injury (ABI) resulting from idiopathic cerebral hemorrhage in the left temporal lobe during the first week of life. Injury was diagnosed by a neurologist following a CT scan ordered after the patient suffered a grand mal seizure. Damage to the hypothalamus was also noted. At the intake interview, the patient complained of slow processing speed, problems with visual and auditory input, depression, poor work and driving performance, and lack of independence. He described his life as, “so overwhelming: people, noise, things moving so fast, so exhausting. Feels like I have to concentrate so hard just to keep up.” During the study period, his cognitive trainer noted improvement with working and long-term memory. The client concurred with improvements in memory and processing, especially remembering names and driving directions. After completing 60 h of training, he reported increased confidence, independence, outlook, new social identity, and multiple quality-of-life improvements, such as living on his own and employment gains. His mother stated, “He’s a different person – smiles more, conversational, more extroverted. Not the shy, introverted [young man] anymore.”

#### Patient Six

Patient six was a 15 years old male who suffered a concussion due to a fall from a shopping cart as a toddler. There was no loss of consciousness. He was diagnosed following a CT scan by the emergency room physician. At the beginning of the study, he presented with poor school performance, distractibility, and behavioral problems. His mother reported, “He is so smart – but doesn’t do well in school. Lots of trouble remembering things – we do lots of lists, have to keep him organized.” The client stated a desire to feel less stressed in school and improve his academic achievement. Following 60 h of cognitive training, he reported improved memory, focus, relationships, and school achievement. He stated, “I definitely pay attention more…when my mom tells me to do something, I don’t forget it. History was really hard for me before – now it’s easier!”

### Individual Psychometric Testing Results

Table 1 illustrates the clinically significant change analysis results and Table 2 indicates the magnitude of each change. All six patients (100%) achieved significant clinical change and significant Reliable Change Index on GIA (or IQ) score, indicating overall recovery effects from the intervention.

**TABLE 1:** Cut score thresholds and clinically significant change in Woodcock Johnson W scores.

Construct	Cut score	Patient 1		Patient 2		Patient 3		Patient 4		Patient 5		Patient 6	
		Pre	Post	Pre	Post	Pre	Post	Pre	Post	Pre	Post	Pre	Post
IQ score	521	511	530*	521	532*	523	535*	523	538*	530	539*	525	538*
Long-term memory	503	491	509*	509	514*	513	516*	506	511*	491	515*	519	527*
Visual processing	510	514	532*	508	505	500	539*	510	514*	522	532*	522	536*
Auditory processing	515	508	523*	520	531*	517	530*	522	538*	535	539*	504	508
Fluid Reasoning	524	545	549*	501	538*	528	534*	521	545*	549	555*	541	563*
Processing speed	543	510	530*	534	561*	554	558*	544	554*	540	552*	560	567*
Working memory	518	465	524*	543	538	539	547*	516	531*	531	543*	520	543*

*Post-test scores met cut score threshold for clinically significant change.

**TABLE 2:** Magnitude of change by patient and construct.

Construct	Patient 1	Patient 2	Patient 3	Patient 4	Patient 5	Patient 6
IQ score	9.2*R	5.9*R	6.4*R	7.7*R	4.5*R	6.2*R
Long-term memory	6.3*R	2.0*R	1.3U	2.2*R	9.3*R	3.1*R
Visual processing	3.9*R	−0.69U	8.5*R	0.88U	2.0*R	2.9*R
Auditory processing	3.2*R	2.4*R	2.7*R	3.4*R	0.89U	0.83U
Fluid Reasoning	0.60U	6.9*R	1.1U	4.1*R	0.90U	3.7*R
Processing speed	5.7*I	7.7*R	1.4U	7.1*R	8.6*R	2.1*R
Working memory	10.2*R	−1.1U	1.4U	3.1*R	1.9*R	4.8*R

*Significant reliable change index > 1.96. R, recovered; I, improved; U, unchanged.

In addition to the composite IQ scores, 34 of the 36 (94%) subtest score changes were clinically significant, and 25 scores (69%) revealed recovery or improvement across participants. Eleven scores (30%) remained clinically unchanged, but none deteriorated from pretest levels. The rates of recovery on individual subtest scores ranged from 50 to 87%. In long-term memory, 83% (five of six patients) showed recovery. In visual processing, auditory processing, processing speed, and working memory, 67% (four of six patients) showed recovery. In fluid reasoning, 50% (three of six patients) showed recovery.

### Group Psychometric Testing and Functional Rating Results

Wilcoxon results for the PCRS components at pre-intervention and post-intervention are presented in Table 3. Pre-intervention Activities of Daily Living (ADL) ratings, cognition ratings, and overall impairment ratings were considered mild, on average. The interpersonal/emotional impairment ratings were moderate. Both self and family member ADL ratings showed patients improved in their activities in daily living – improving, on average, from “mild impairment” to “no impairment.” The growth was statistically significant for differences in self reporting but not as reported by others. On the measure for interpersonal/emotional impairment, patients improved significantly from “moderate impairment” to “mild impairment” as reported by the participants and by others.

**TABLE 3:** Statistical analysis results for Patient Competency Rating Scale (PCRS).

Area of rating	Pre			Post			Change	
	*M*	*SD*	Median	*M*	*SD*	Median	*Z*	*P*
**Self-ratings**								
Activities of daily living	27.42	4.05	26.50	32.67	1.21	32.00	–2.21	0.03
Interpersonal/emotional	34.17	9.58	34.50	44.92	11.48	48.50	–2.20	0.03
Cognition	32.50	7.92	33.50	39.92	7.49	42.50	–2.21	0.03
Overall impairment	93.08	19.69	90.75	116.33	20.29	123.00	–2.20	0.03
**Family member ratings**								
Activities of daily living	28.25	3.90	28.50	30.00	4.73	31.50	–1.48	0.14
Interpersonal/emotional	32.67	6.74	32.50	42.33	10.31	47.50	–2.20	0.03
Cognition	32.92	4.04	31.25	39.00	7.27	42.00	–1.57	0.12
Overall impairment	93.83	11.57	93.75	111.33	20.26	122.50	–2.20	0.03

*Significant differences (p < 0.05).

On the cognition metric, scores showed increases on self-reports and by others, but the scores did not demonstrate an improvement in impairment classification, remaining at “mild impairment.” Moreover, the increases were statistically significant only on the self-reports. Similarly, the overall composite scores showed significant increases as reported by self and others, but this did not translate into improvements in impairment classification, which remained at “mild impairment.”

We also examined Woodcock Johnson IV sub-tests from pre- to post-intervention. These included standard scores for visual processing, auditory processing, fluid reasoning, processing speed, working memory, long term memory, and general intelligence (GIA). As Table 4 indicates, participants saw, on average, mean and median increases on all tests, and standard deviations narrowed for all tests except for visual and auditory processing. The greatest mean difference was evident for General Intellectual Ability (Δ = 20 points), and the smallest was processing speed (Δ = 8.5 points). On three of the sub-tests (long term memory, visual processing, and general intelligence), growth exceeded one standard deviation, and on fluid reasoning growth was almost one standard deviation. Moreover, all differences except working memory and visual processing were statistically significant at *p* < 0.05.

**TABLE 4:** Results for Woodcock Johnson IV test standard scores.

Cognitive skill	Pre			Post			Change	
	*M*	*SD*	Median	*M*	*SD*	Median	*Z*	*p*
Working memory	103.00	27.83	108.50	116.67	13.94	118.50	–1.15	0.25
Long-term memory	105.00	20.91	108.50	123.83	12.70	121.00	–2.21	0.03
Visual processing	107.67	10.61	106.00	125.17	19.13	131.00	–1.78	0.08
Auditory processing	108.33	14.00	110.50	119.00	14.77	120.50	–2.20	0.03
Processing speed	97.33	12.60	95.50	105.83	9.02	107.00	–2.20	0.03
Fluid reasoning	111.67	15.93	116.00	126.17	10.30	126.00	–2.23	0.03
General intellectual ability	108.33	9.52	110.50	128.50	6.66	131.00	–2.20	0.03

*Significant differences (p < 0.05).

**FIGURE 2:**
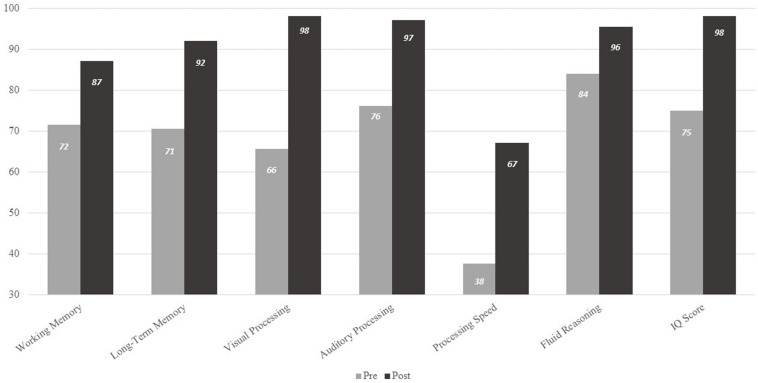
Pre- and post-percentiles on the Woodcock Johnson IV.

Finally, Figure 2 presents the WJ IV test results in percentiles. All scores showed growth. On five of the seven sub-tests, post-test scores exceeded the 90th percentile. Another – working memory – approached the 90th percentile. Processing speed showed substantial percentile growth as well.

### Qualitative Results

#### Cross-Case Synthesis

Table 5 details the synthesis of qualitative findings from the five patients including self-reported pre-intervention symptoms and self-reported post-intervention outcomes. All six patients disclosed social/relational struggles prior to the training protocol, and all six patients commented on improvements in this area post-training. Additionally, every patient described improvements in mood from pre to post-intervention. Three of the six patients reported problems with driving pre-intervention, and all three recorded gains in or recovery of driving ability post-training.

**TABLE 5:** Cross-case synthesis of qualitative outcomes.

	Self-reported pre-intervention symptoms	Self-reported post-intervention outcomes
Patient one	overwhelmed by visual/auditory input, memory loss; tearfulness, independence decline; loss of social connections; reading inefficiency (hobby); not driving	better focus/attention, memory, and visual/auditory processing; more confident; renewal of social identity; able to read for hobby again; resumed driving and cooking
Patient two	lack of focus; depressed and anxious; no social interaction; unemployed; not driving	improved focus and memory; hopeful, setting goals; new friends; new job; now driving
Patient three	poor focus, memory, processing speed; impulsivity and anger; social difficulty and fighting; avoidance of schoolwork	less distracted, improved concentration and memory, faster processing; more motivation and goal setting, positive outlook; less argumentative, new social identity development; improved schoolwork, hobbies, sports
Patient four	memory and focus lapses, poor visual/auditory processing; depression, lack of motivation; poor social identity; low school achievement	better memory, increased attention, processing speed and visual/auditory processing; more confidence and perseverance, positive outlook; improved social conversational skills; better school performance
Patient five	slow processing speed, easily overwhelmed by visual/auditory input; sometimes depressed; poor work performance; driving difficulties, still living with parents	improved memory, faster and more efficient processing; more confident, independent, and outgoing; novel social identity; new work achievements; driving skills improved, achieved independent living
Patient six	distractibility; anxious and emotionally volatile; negative socialization experiences; low school achievement	improved focus and memory; more cooperative, calmer; improved social relationships; better school performance

#### Qualitative Thematic Analysis

Through the inductive approach to thematic analysis (Liu, 2016), several themes emerged: *Cognition, Mood, Social Identity*, *Performance*, and *Instrumental Activities of Daily Living (IADLs*). During pre-intervention interviews the most prevalent symptoms reported were cognitive complaints, poor or negative mood, and weak social identity. Correspondingly, the most common post-intervention outcomes were regarding improved cognitive efficiency (including memory, attention, and processing speed), a more positive mood, and enriched social identity (including better relationships and communication skills). The comprehensive synthesis of pre-intervention and post-intervention comments from patients is illustrated in Table 6.

**TABLE 6:** Qualitative thematic analysis; themes in order of prevalence.

Pre-intervention most frequently cited symptoms	***Cognition:*** memory lapses, inability to focus, slow processing, reading/listening problems	***Mood:*** depression, anger, anxiety, lack of confidence, low energy, apathy for the future	***Social Identity:*** poor or impaired communication, little or no social connectedness, troubled interpersonal relationships	***Performance:*** work difficulty/unemployment, poor school achievement, hobby/sport frustrations	***IADL:*** diminished driving ability, inadequate self-care, financial, and/or household maintenance
Post-intervention most frequently cited outcomes	***Cognition:*** improved memory, better focus/attention, faster processing, enhanced reading or visual/auditory processing	***Mood:*** improved outlook, reduced anxiety, more confidence, diligence, higher energy, goal setting	***Social Identity:*** enriched communication, increased social connectedness, better interpersonal relationships	***Performance:*** improved work outcomes, better school achievement, increased hobby/sport satisfaction	***IADL:*** ameliorated driving ability, improved self-care, financial and household management

### Imaging Results

**FIGURE 3:**
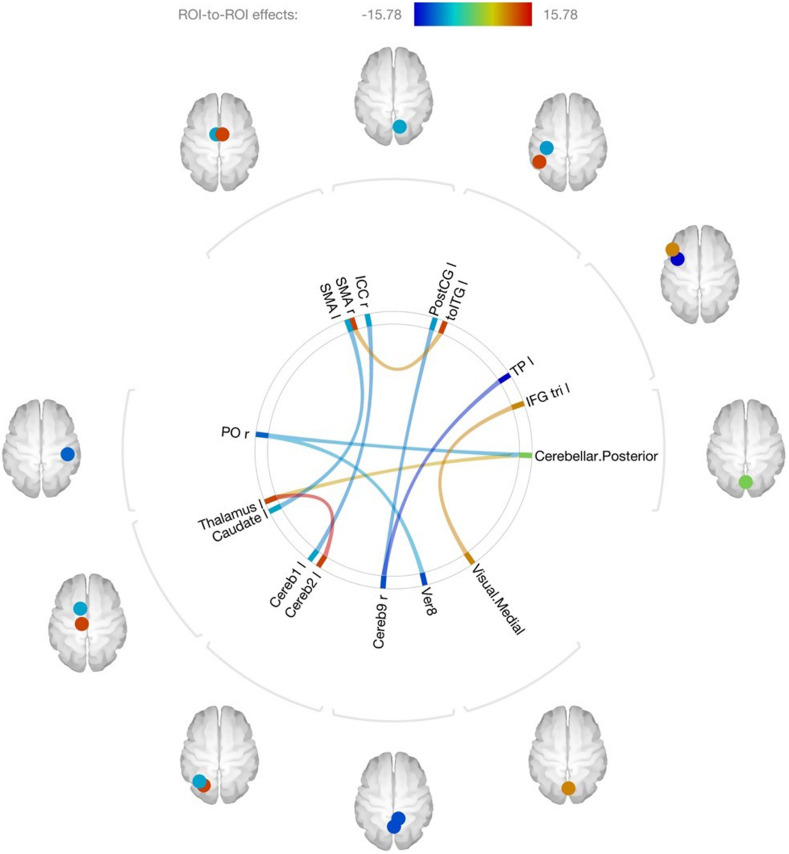
Connectome map of changes in resting state connectivity after training, ROI-to-ROI analysis. SMA, supplementary motor area; ICC, intracalcarine cortex; PostCG, post-central gyrus; toITG, inferior temporal gyrus, temporooccipital part; TP, temporal pole; IFG tri, inferior frontal gyrus, pars triangularis; Ver8, vermis Lobe 8; Cereb9, cerebellum lobe 9; Cereb2, cerebellum lobe VIIA, crus 2; Cereb1, cerebellum lobe VIIA, crus 1; PO, parietal operculum; r, right; l, left.

For the second-level region-of-interest (ROI) to ROI analysis of 164 regions, 10 FDR-corrected (*p* < 0.050, seed-level correction, two-sided) significant changes in resting state functional connectivity at post-intervention were identified. These changes are illustrated in Figure 3 and outlined in Table 7. Seven of the 10 changes involved the cerebellum or cerebellar networks, two involved the motor cortex, and one involved the visual network. Of the significant changes in pre-/post-resting state functional connectivity, two were associated with an increase in connectivity: left Crus 1 of the cerebellum with the right intracalcarine cortex (aka primary visual cortex) and right lobe 9 of the cerebellum with the left post-central gyrus (aka somatosensory cortex).

**TABLE 7:** Changes in resting state connectivity after training, ROI-to-ROI analysis.

Region-of-interest to region-of-interest	*t*	β	p-FDR	Interpretation
Left crus 1 of the cerebellum, right intracalcarine cortex	–9.5	–0.12	0.035	Increase in anticorrelation, increase in connectivity
Right lobe 9 of the cerebellum, left postcentral gyrus	–9.7	–0.13	0.016	Increase in anticorrelation, increase in connectivity
Right lobe 9 of the cerebellum, left temporal pole	–14.2	–0.26	0.005	Conversion to anticorrelation, decrease in connectivity
Vermis 8 of cerebellum, right parietal operculum cortex	–8.0	–0.20	0.040	Conversion to anticorrelation, decrease in connectivity
Left crus 2 of the cerebellum, left thalamus	15.8	0.21	0.003	Conversion to correlation, decrease in connectivity
Posterior cerebellar networks, right parietal operculum cortex	–8.4	–0.17	0.049	Conversion to anticorrelation, decrease in connectivity
Posterior cerebellar networks, left thalamus	7.6	0.20	0.049	Conversion to correlation, decrease in connectivity
Left supplementary motor cortex, left caudate	–9.1	–0.12	0.044	Decrease in correlation, decrease in connectivity
Right supplementary motor cortex, left inferior temporal gyrus	9.0	0.21	0.046	Conversion to correlation, decrease in connectivity
Medial visual network, left inferior frontal gyrus	9.4	0.15	0.037	Decrease in anticorrelation, decrease in connectivity

For the DTI structural connectivity Fractional Anisotropy (FA) analysis, we found three significant positive correlations out of the 17 total correlations between changes in IQ score and changes in white matter tract FA after controlling for multiple comparisons using the Benjamini Hochberg FDR method. The significant correlations are shown in Table 8.

**TABLE 8:** Significant correlations between changes in fractional anisotropy (FA) index and GIA score.

White matter fiber tract	*r*	*p*-value	Rank	BH critical value
Right corticospinal tract	0.952*	0.003	1	0.012
Left uncinate fasciculus	0.897*	0.015	2	0.024
Left corticospinal tract	0.850*	0.032	3	0.035

*r is the correlation of change in FA to change in GIA score; ^∗^significant at p < Benjamini Hochberg (BH) Critical Value.*

## Discussion

The aims of the current study were (a) to explore cognitive and functional improvements following 60 h of clinician-delivered cognitive training for six patients with mild brain and (b) to use MRI to explore the mechanism of change in cognitive training. We found significant changes across all outcome areas examined, including objective cognitive test scores, subjective functional ratings, resting-state connectivity, and objective neural correlates of cognitive change.

### Cognitive Training, Cognition, and Behavior

We examined cognitive and functional outcomes following cognitive training. As a group, all WJ IV cognitive test scores showed improvement with statistically significance changes on five of the seven measures (long-term memory, processing speed, reasoning, auditory processing, and overall IQ score). The mean change in IQ score was 20 points, from a mean of 108 to a mean of 128. The clinical significance of individual cognitive change was indeed robust. All six patients could be classified as “recovered” based on the significant analysis of change in their overall IQ scores, a global composite indicator of cognitive function. Our finding is consistent with our prior research on the same cognitive training method studied for soldiers with moderate to severe TBI (Ledbetter et al., 2017). This suggests *Brain Booster* by LearningRx has potential to remediate cognitive skills across various brain injury causes and levels of severity. An encouraging result is the clinical significance of the changes on 94% of the individual subtests that measured multiple cognitive skills, suggesting the training tasks effectively targeted each skill or the training transferred to other tested skills for this group of patients.

The functional changes were equally as encouraging. At the pre-intervention stage, Activities of Daily Living (ADL) ratings, cognition ratings, and overall impairment ratings were considered mild on average. The interpersonal/emotional impairment ratings were moderate. Statistically significant improvements were noted in all four sets of impairment ratings completed by the patients themselves, and significant improvements in two of the four categories (interpersonal/emotional and overall impairment) were noted in the ratings completed by a significant other. Both self and family member ADL ratings showed patients improved in their activities in daily living – improving in classification from “mild impairment” to “no impairment.” Patients also improved significantly in interpersonal/emotional impairment from “moderate impairment” to “mild impairment” as reported by both the patients and family members.

The functional improvements noted on the rating scales were echoed in the thematic analysis of interview data. Five themes emerged from the analysis including improvements in cognition, mood, social identity, performance, and Instrumental Activities of Daily Living (IADLs). Prior to the intervention, the most prevalent symptoms reported were cognitive complaints, negative mood, and a weak social identity including relationship struggles. Subsequently, the most common post-intervention outcomes reported related to improvements in the same three areas. In addition, half of the patients reported problems with driving that resolved following the intervention. These quality of life findings are consistent with our previous research on the same intervention with both children and adults in which we found transfer to improvements in relationships, self-confidence, cognition, work and school performance, and daily life skills (Ledbetter et al., 2017; Moore et al., 2018, 2019; James et al., 2019). While this study did not attempt to statistically correlate the cognitive and behavioral variables, these trends corroborate current research on the relationship between cognitive and social outcomes (Kuiper et al., 2016). There can be positive behavioral effects from the therapeutic relationship regardless of the nature of the intervention. That is, the significant attention given to each patient during the study could contribute to the improvement in mood and the subsequent improvement in cognition and IADLs/ADLs. Although prior research on the impact of mood on cognition in people without clinical depression suggests mood does not have a significant influence on general cognitive function (Chepenik et al., 2007), it would be interesting to quantify that relationship in future studies.

### Cognitive Training and Brain Connectivity

Previous fMRI research with TBI patients revealed disruptions in functional network connections in the brain; and that cognitive training is associated with improvement in connectivity (Han et al., 2017, 2018a, 2020). The current study findings are consistent with this previous work. We documented ten significant region-to-region changes in connectivity following cognitive training. It was interesting to find changes involving the cerebellum, which is typically associated with motor control. However, there is a robust body of research now pointing to cerebellar function in higher thinking. For example, the Crus 1 has been linked to language, verbal working memory, and executive function tasks (Stoodley and Schmahmann, 2009); and the vermis has been associated with affect and voluntary control of emotion processing (Schmahmann, 2004). Indeed, our qualitative outcomes are consistent with these prior associations. Future studies with a larger sample, a control group, and optimized methods for studying the cerebellum would allow for more in depth analysis of the role of role of the cerebellum in cognitive training.

Prior research indicates there may be reduced white matter integrity in the brain following a TBI as measured by the Fractional Anisotropy (FA) Index (Kim et al., 2019). This may be a result of demyelination or a reduction in fiber density in the white matter tracts. In the current study, we explored whether changes in the FA Index correlated with changes in overall IQ score at post-test and found three significant correlations between change in IQ score and change in the white matter integrity of both left and right CST as well as the left uncinate fasciculus. The corticospinal tract is predominately associated with motor function and sensory processing, so we find it intriguing that cognitive improvements correlated with increased integrity of the CST. However, there is a motor component to the intervention with a strong eye-hand coordination element. Trainers noted functional improvement in motoric coordination in all six patients by the end of the study period, which may help explain the CST correlation with cognitive improvement. Further, the uncinate fasciculus has a variety of functions including connecting the limbic system to the orbital frontal cortex (Von Der Heide et al., 2013). Our results are consistent with other recent studies showing correlations between FA and intelligence (Watson et al., 2018; Holleran et al., 2020). Measures of cognitive function, including working memory, have been correlated with FA in the uncinate fasciculus in dementia (Morikawa et al., 2010). Furthermore, the changes we saw in FA in the uncinate fasciculus following training are consistent with cognitive training results in Autism Spectrum Disorder (Pardini et al., 2012). It will be important to explore this association with a larger sample in future research before any conclusions can be drawn about the mechanism(s) driving training-induced correlations in brain injury.

### Mechanism of Change

The mechanism of change in cognitive training research such as this is grounded in the concept of neuroplasticity and the prior documentation of experience-induced changes in cortical functioning (see Buonomano and Merzenich, 1998; Schwartz and Begley, 2003; Huang, 2009). Prior evidence of functional map expansion following LearningRx training (Ledbetter et al., 2016; James et al., 2019) supports our proposed mechanism of change through training-induced plasticity for the current study as well. However, we do not know if this training-induced neuroplasticity is the result of increased synaptic activity, restoration of damaged connections, and/or from neural reassignment. This is indeed an on-going debate in the neuroimaging field (Ernst and Frisén, 2015; Sorrells et al., 2018) and well beyond the scope of this study. With a small sample, we are currently not able to elucidate a definitive mechanism of change but simply identify trends in data that support further investigation and support the use of functional and structural neuroimaging with MRI for this investigation.

### Limitations and Future Research

There are several limitations worth discussing. First, as a multiple case study, the sample size was small, which limits the ability to extrapolate the findings beyond the current study. In future research, it will be important to include a larger sample size with a homogenous group to increase statistical power and generalizability. However, the heterogeneous nature of this sample shows the applicability of the intervention across ages from teens, 20s, and 30s, as well as applicability of the intervention across times since injury. Further, guidance about case selection in multiple case study research suggests cases should reflect how diverse the phenomenon under study can be in the population and should provide the ability to examine both similarities and differences across cases (Mills et al., 2010).

Another limitation of this study is the lack of a control condition. Future research should include a control group in order to make causal rather than correlation conclusions about the impact of the intervention. A design with three sets of matched controls (a no training homogenous brain injury group and two non-injury groups, one receiving training and one not receiving training) would strengthen the design even further. Next, the use of self-reports and interviews always risks an expectancy effect, or the patient’s hope/belief that he or she will improve from the intervention. Without a control group, there’s no way to mitigate this risk in the current study. Finally, the current study does not address follow-up assessment on any of the measures. Future research should examine the long-term effects of the intervention. Despite these significant limitations, the use of objective tests and neuroimaging reduces the impact of an expectancy effect, particularly when improvements are found on these robust objective measures as well. Further, the use of within-subjects data analysis techniques certainly adds to the credibility of the case study approach, and a case study enables detailed exploration of the phenomenon to better inform the design of future studies – which was a primary goal of this work.

## Conclusion

This study adds to the growing body of literature examining the effects of cognitive training for mild brain injury and to the collection of research on the benefits of cognitive training in general. Although the purpose of the current study was to explore changes in cognition and daily functioning following 60 h of cognitive training with *Brain Booster* for patients with mild brain injury, we noted some interesting neuroimaging results in this this preliminary study that will support a larger controlled study. The clinically significant changes in overall IQ score and individual cognitive skills were robust and consistent with prior within-subject outcomes on the same intervention (Ledbetter et al., 2017; Moore et al., 2018). Further, the significant correlations between IQ score changes and changes in structural white matter integrity suggest there may indeed be neural correlates to cognitive training change – an encouraging finding in a small sample supporting experience-based cortical plasticity that begs to be studied on a larger scale. We hope this study continues the discussion about the promise of clinician-delivered cognitive training for those recovering from brain injury and sparks new ideas for continued research in the area of cognitive training specifically with the mild brain injury population.

## Data Availability Statement

The datasets generated for this study are available on request to the corresponding author.

## Ethics Statement

The studies involving human participants were reviewed and approved by Gibson Institute of Cognitive Research Institutional Review Board (IRB). Written informed consent to participate in this study was provided by the participants’ legal guardian/next of kin. Written informed consent was obtained from the individual(s) and/or minor(s) parent for the publication of any potentially identifiable images or data included in this article.

## Author Contributions

AM acted in the capacity of primary investigator, supervised the neuropsychological assessments and cognitive training portions of the intervention, and drafted the manuscript. DC conducted the quantitative data analyses and edited the manuscript. TM conducted the qualitative data collection and analysis, coordinated the patient schedules, and drafted the literature review portion of the manuscript. CL acted as co-PI, oversaw the MRI acquisitions, conducted the MRI data analyses, and edited the manuscript. ED co-conducted and interpreted the MRI data analysis and edited the manuscript. JM served as the intervention coordinator and edited the manuscript. RJ served as the study physician, validated patient history and eligibility, assisted with recruitment, and edited the manuscript. All authors contributed to the article and approved the submitted version.

## Conflict of Interest

AM and TM were employed by the non-profit research institute associated with the intervention used in the current study but have no financial interest in the outcomes of the research. JM currently volunteers on the research institute’s non-profit board for no financial renumeration but did not have that role at the time of the study. CL serves on the scientific advisory board for the intervention used in the study but receives no financial renumeration for such volunteer service. The remaining authors declare that the research was conducted in the absence of any commercial or financial relationships that could be construed as a potential conflict of interest.

## Footnotes

1 www.nitrc.org/projects/conn

2 http://surfer.nmr.mgh.harvard.edu/
